# The Efficacy of Cognitive Training for Elderly Chinese Individuals with Mild Cognitive Impairment

**DOI:** 10.1155/2019/4347281

**Published:** 2019-11-30

**Authors:** Zhenren Peng, Hu Jiang, Xiaomin Wang, Kaiyong Huang, Yukun Zuo, Xiangmin Wu, Abu S. Abdullah, Li Yang

**Affiliations:** ^1^Department of Occupational and Environmental Health, School of Public Health, Guangxi Medical University, Nanning 530021, China; ^2^Department of Epidemiology and Health Statistics, School of Public Health, Guangxi Medical University, Nanning 530021, China; ^3^Global Health Research Center, Duke Kunshan University, Kunshan 215316, China; ^4^Boston University School of Medicine, Boston Medical Center, Boston, MA 02118, USA

## Abstract

The age of the population is shifting toward the elderly range, which may lead to an increased risk of mild cognitive impairment (MCI). The aims of this study are to evaluate the cognitive function in elderly people using the Montreal Cognitive Assessment (MoCA), to identify the relationship between cognitive function and different characteristics, and to evaluate the efficacy of the intervention after six months of cognitive training. In this study, we included 2886 subjects aged ≧60 years in the baseline survey, and 140 subjects with MCI who participated in the baseline survey were randomly divided into an intervention group (*N* = 70) and a control group (*N* = 70). The control group was not provided any intervention measures, and the intervention group was administered cognitive training. The education level, monthly income, sleep time, exercise time, reading times, and time spent engaging in community activities and performing housework were positively correlated with MoCA scores, but age was negatively correlated with MoCA scores. The total MoCA score of the intervention group increased from 19.77 ± 2.24 points to 21.09 ± 2.20 points after six months of cognitive training, but the score of the control group decreased from 20.41 ± 2.10 points to 19.17 ± 2.57 points. The two-way repeated-measures ANOVA revealed a very significant effect of the interaction between time and cognitive training on the total MoCA score. Seventeen participants in the intervention group improved to normal levels, and no participants progressed to dementia after six months of cognitive training. Thus, the efficacy of the intervention was statistically significant. Our study concludes that older age is associated with a cognitive decline. Factors that are more likely to protect against cognitive decline included a higher education level and monthly income, sufficient sleep time, regular physical exercise and reading, frequently engaging in community activities, and continuing to perform housework. Moreover, the cognitive training intervention is effective and may help to decrease the deterioration of cognitive function in patients with MCI, and the interaction between intervention time and cognitive training significantly improves cognitive function.

## 1. Introduction

The pace of population aging is increasing dramatically worldwide, with many social, economic, and health implications. The global population aged greater than 60 years is expected to increase from 605 million in 2000 to 1.2 billion by 2025 and to 2 billion by 2050; approximately two-thirds of these older people live in low-income and middle-income countries (LMICs) [1]. The rapid growth of the elderly population in China, which will exceed 400 million by 2033, will represent the largest number of elderly individuals in any country in the world [1]. This growing number of older individuals will lead to an increase in the incidence of aging-related disorders such as mild cognitive impairment (MCI) in older populations [2], underscoring the need for innovative programs to prevent MCI [3].

MCI is a clinical condition characterized by a reduction in memory and/or other cognitive processes that are insufficiently severe to be diagnosed as dementia but are more pronounced than the cognitive decline associated with normal aging [4]. MCI is an intermediate state between normal cognition and dementia, with essentially preserved functional abilities [5]. Therefore, this condition is easily underestimated [6]. The diagnosis of MCI is based mainly on the patient's history and a cognitive examination [7], and the Montreal Cognitive Assessment (MoCA) is a useful instrument to detect mild impairments in cognition and has become a common tool used to diagnose both MCI and dementia [8]. The global prevalence of MCI in the population aged ≧60 years is up to 38.60% [9], but it is approximately 11.00–20.00% among older Chinese adults [1, 10, 11]. MCI is a risk factor for dementia [12] and is associated with a 6-fold increased risk of Alzheimer's disease (AD) [13]. Additionally, more than 50.00% of people with MCI subsequently develop dementia [14].

Over the past few decades, the identification of the factors predicting cognitive decline has become increasingly important in the field of geriatrics [15] to facilitate targeted interventions. Researchers anticipate that the detection of cognitive decline and delivery of interventions to at-risk individuals at this earliest stage may prove more effective in preserving cognitive function [15]. However, the existing reviews and previous meta-analyses have reported varying findings concerning the benefits of cognitive training. While several studies reported the benefits of cognitive training [16–19], others have found little or no advantage [20–23]. Several reviews report a benefit of cognitive strategies. In a recent study, the practice of 6 months of dancing training positively affected body composition and increased fitness performance, memory functions, and anxiety in elderly people [24]. We hypothesized that cognitive training might improve cognitive functions and delay the progression from MCI to AD. We evaluated the cognitive function of elderly people using MoCA scores, as no published studies among Chinese populations have used the MoCA scores of elderly people to evaluate the effect of cognitive training. The aims of our study are to evaluate the cognitive function of elderly people using the Beijing version of the Montreal Cognitive Assessment (MoCA-BJ) [25] and then to identify whether cognitive function correlates with different characteristics. In addition, we aim at evaluating the efficacy of the intervention after three and six months of cognitive training.

## 2. Materials and Methods

### 2.1. Design

In the baseline survey, the study used the cluster sampling method to select 3000 subjects from three communities in Nanning, Guangxi, China. All subjects were selected from community-dwelling elderly people that were notified on the telephone by community health workers from July to September of 2017. A structured interviewer-administered questionnaire was used to collect data on sociodemographics and lifestyle factors for the subjects; MoCA-BJ [25] was used to evaluate the cognitive function. The evaluation included 7 cognitive domains: visuospatial and executive function (trail-making B task (1 point), cube copy (1 point), and clock-drawing (3 points)), naming (3 points), attention (forward/backward digit span (2 points), vigilance/tapping (1 point), and serial 7 subtraction (3 points)), language ((sentence repetition (2 points) and verbal fluency (1 point)), abstraction (the 2-item verbal abstraction, total of 2 points), delayed recall/short-term memory (5 points), and orientation (6 points) [25, 26]. The total score of MoCA-BJ [25] was 30 points.

We conducted an open-label randomized controlled trial among 140 subjects with MCI who were randomly selected from the baseline survey within a community. Selected subjects were randomly assigned to the intervention group (*N* = 70) and the control group (*N* = 70). The control group was not provided any intervention measures. The cognitive training was controlled and executed by the designer, and it was conducted from May to December of 2018. The study used the group training method for the intervention group, and the participants in the intervention group all gathered in a classroom and were provided cognitive training every two weeks for six months by the designer; each training session lasted approximately 90 minutes. The cognitive training intervention included memory training, attention training, and calculation training. Memory training included seven-piece board recovery training, picture-reading memory, reading aloud, and reciting phrases; attention training included colour reaction training and Schulte Grid training; and calculation training included two simple calculation questions and one simple application question for calculation in each intervention process. Then, the participants in the intervention and control groups were evaluated using the MoCA-BJ [25] by investigators at the third and sixth months.

Participation in the study was voluntary; individuals who agreed to participate signed an informed consent form. The study protocol was approved by the Ethics Committee of Guangxi Medical University.

### 2.2. Sample Size Calculation

Because the baseline survey was a cross-sectional survey, the sample calculation formula (*N*=*D* × (*Uα*^2^ × *π* × (1 − *π*)/*δ*^2^)) was used to calculate sample size in this study. The lowest prevalence of MCI in elderly individuals aged ≧60 years was reported to be approximately 5.00% [27]. If *α* = 0.05, *u*_*α*_ = 1.96, the overall prevalence (л) = 5.00%, the error tolerance (*δ*) = 1.00%, and the accuracy of survey (*D*) = 1.50, the required sample size would be 2766 using the formula listed above. In this study, considering the rates of loss to follow-up, nonrespondents, and invalid questionnaires, the sample size was increased to 3000.

The sample sizes of the intervention and control groups were calculated using G-Power 3.1.9.4 (Kiel University, Kiel, Germany). According to the study by Anderson-Hanley et al. [28], the average and standard deviation of MoCA scores for the intervention and control groups are 24.70 ± 3.56 and 23.20 ± 4.97 points, respectively. Therefore, we substituted the average and standard deviation of MoCA scores reported in the study by Anderson-Hanley et al. [28] into G-Power 3.1.9.4 to calculate the validity. The validity of the intervention and control groups was 0.726 and 0.347, respectively, and the intermediate validity was 0.536. Based on a validity = 0.536, *α* = 0.05, *u*_*α*_ = 1.96, *β* = 0.20, and power of the tests (1 − *β*) = 0.80, we used the independent-samples *t* test to calculate the sample size with G-Power 3.1.9.4, and the sample sizes of the intervention and control groups were 56 and 56, respectively. In this study, considering the rates of loss to follow-up and nonrespondents, the sample sizes of the intervention and control groups were increased to 70 and 70, respectively.

### 2.3. Sample Inclusion and Exclusion Criteria

In the baseline survey, subjects aged ≧60 years who had lived in Nanning for more than six months and were fluent in local dialects were all able to be included in this study. Subjects suffering from the following diseases were excluded: brain tumours, Parkinson's disease, unstable internal medical diseases that could influence brain function or cognitive function, a history of acute cerebrovascular disease within three months, active epilepsy, dementia, severe sensory impairment, or a history of mental illness.

When selecting the participants for the intervention and control groups, subjects aged ≧60 years who had lived in Nanning for more than six months, were fluent in the local language, and had complained of memory loss or the decreased abilities of daily living or were diagnosed with MCI in the baseline survey were all included in this study. Subjects who suffered from the following diseases were excluded: patients with psychiatric disorders or who were not fluent in the local language, patients with serious somatic diseases or severe sensory disorders, or patients who had been diagnosed with dementia and neurological disorders in the baseline survey. Additional exclusion criteria were patients who were unable to continue to participate in the intervention process for personal or family reasons or withdrew by themselves, patients who did not participate in the intervention process for three or more sessions, or patients who experienced other serious illnesses in the intervention process.

### 2.4. Diagnostic Criteria for MCI and Dementia

MCI was diagnosed if the subject met the following criteria: memory complaint, normal activities of daily living, normal general cognitive function, abnormal memory for age, and a lack of dementia [29]. The MoCA score is used to diagnose MCI, and the subject is diagnosed with MCI if the MoCA score is ≦23 points [30] or with dementia if the MoCA score is ≦14 points [31]. In addition, because the education level may influence the MoCA score [26], the following criteria were used to diagnose MCI in this study: the optimal number of points is <17 for illiterate individuals, <20 for individuals with 1 to 6 years of education, and ≦23 for individuals with 7 or more years of education [32].

### 2.5. Statistical Analysis

All data were entered by double entry using EpiData 3.0 and were analysed using IBM SPSS 23.0. In the baseline survey, a *t*-test or one-way ANOVA was used to analyse differences in MoCA scores in subgroups stratified by demographic characteristics, and the Spearman correlation analysis was used to analyse whether MoCA scores correlated with different demographic characteristics. For the intervention study, the outcome variables were analysed using two-way repeated-measures ANOVA. The within-subject factor was the time (baseline and 3 and 6 months after the intervention), and the between-subject factor was the cognitive training (intervention or control group). Post hoc pairwise comparisons with the Bonferroni adjustment were applied when significant interaction effects were observed. The Greenhouse–Geisser correction was used when Mauchly's test of sphericity was violated. Partial eta-squared (*ηp*^2^) values were reported to confirm the effect size in the two-way repeated-measures ANOVA tests. Moreover, the chi-square test was used to evaluate the effect of the intervention on MoCA scores by assessing the prevalence of participants whose score improved to normal, decreased to dementia, or did not change. Statistical significance was established at *p* < 0.05.

## 3. Results

### 3.1. Basic Information Obtained from the Baseline Survey

Of the participants included in the baseline survey (*N* = 2886), 58% were male and the mean age of the participants was 68.99 ± 5.92 years. Notably, 28.86% (833/2886) of the participants were diagnosed with MCI, 8.49% (245/2886) with dementia, and 7.31% (211/2886) with neurological diseases.

### 3.2. MoCA Scores of the Baseline Survey

Table 1 summarizes the MoCA scores. The total MoCA score was 21.69 ± 4.64 points.

### 3.3. Distribution of MoCA Scores according to Demographic Characteristics

The results for the MoCA scores in subgroups stratified by different demographic characteristics are shown in Table 2. According to the *t*-test or one-way ANOVA, MoCA scores were statistically significantly associated with gender (*p* < 0.001), age (*p* < 0.001), education level (*p* < 0.001), monthly income (*p* < 0.001), sleep time (*p* < 0.001), exercise time (*p* < 0.001), reading times (*p* < 0.001), time spent engaging in community activities (*p* < 0.001), and time spent performing housework (*p* < 0.001). Spearman's correlation analysis indicated positive correlations between the education level (*p* < 0.001), monthly income (*p* < 0.001), sleep time (*p* < 0.001), exercise time (*p* < 0.001), reading times (*p* < 0.001), time spent engaging in community activities (*p* < 0.001), and time spent performing housework (*p* < 0.001) with MoCA scores, but age (*p* < 0.001) was negatively correlated with MoCA scores.

### 3.4. Basic Information Obtained from the Intervention and Control Groups

Table 3 summarizes the availability and basic characteristics of subjects at baseline and at 3 and 6 months of follow-up. No significant differences in gender and mean age were observed among the participants in the intervention and control groups at different evaluation points. More subjects in the intervention group were lost to follow-up at 3 months (11 versus 7) and 6 months (16 versus 11) of follow-up.

### 3.5. MoCA Scores of the Intervention and Control Groups

The MoCA scores of the intervention and control groups are shown in Table 4. The total MoCA score of the intervention group increased from 19.77 ± 2.24 points to 20.64 ± 2.49 and 21.09 ± 2.20 points after three and six months of cognitive training, respectively, but the score of the control group decreased from 20.41 ± 2.10 points to 19.40 ± 2.57 and 19.17 ± 2.57 points, respectively. The two-way repeated-measures ANOVA showed very significant effects of the interactions between time and cognitive training on the total MoCA score (*p* < 0.001) and the MoCA scores for attention (*p*=0.001), vigilance (*p*=0.046), language (*p*=0.009), delayed recall (*p*=0.044), and orientation (*p*=0.013). The MoCA scores for vigilance (*p*=0.001), sentence repetition (*p* < 0.001), and verbal fluency (*p*=0.001) differed over time (Table 5).

### 3.6. Efficacy of the Intervention

After 3 months, some participants in the intervention group improved to normal levels (23.73%, 14/59) and none progressed to dementia (0.00%, 0/59), while only one participant in the control group (1.59%, 1/63) improved to normal levels and one progressed to dementia (1.59%, 1/63). After 6 months, some participants in the intervention group improved to normal levels (31.48%, 17/54) and none progressed to dementia (0.00%, 0/54), while only one participant in the control group (1.69%, 1/59) improved to normal levels and three progressed to dementia (5.08%, 3/59). The chi-square test showed the significant effect of cognitive training on converting to normal MoCA scores after 3 and 6 months of intervention (*p* < 0.001; Table 6).

## 4. Discussion

To the best of our knowledge, this study is the first to evaluate the cognitive function of elderly people aged ≧60 years in a community-based setting in Nanning using the MoCA-BJ. In our previous study, the overall prevalence of MCI in the population aged ≧60 years was 27.27% [33], which is higher than in other Chinese studies (11.00%–20.00%) [1, 10, 11] but lower than in the city of Guilin, Guangxi, China (37.00%) [34]. When comparing our findings with other international studies including different criteria for defining MCI performed in Asia, the USA, and Europe, a wide range of the prevalence of MCI from 7.90% to 38.60% [9, 13], 5.60% to 27.65% [35, 36], and 5.00% to 20.00% [37], respectively, emerged. A potential explanation for the difference in these results might be the analysis of different regions and countries using different evaluation tools, methods, or criteria to evaluate MCI. Although the researchers used the same tools and criteria to evaluate MCI in the same country, the test results may vary due to the differences in the sociodemographic characteristics of the participants.

An important factor to consider when selecting a cognitive test is how its performance is influenced by demographic factors, such as age and education level [38], and a 1-point lower MoCA score is associated with a 34.00% increased risk of cognitive decline [39]. According to recent studies, the MoCA score is negatively correlated with age and is significantly higher for younger elderly people [40]. The MoCA score is also positively correlated with the education level (*r* = 0.460–0.660) [41, 42]; these results are consistent with our study. Furthermore, sleep disturbance is prevalent and predicts cognitive decline in older people and in patients with neurodegenerative disorders [43], whereas physical activity [44] and good reading habits [45] are factors that protect against cognitive decline. These results are also consistent with our study. Therefore, maintaining good quality sleep, regular exercise, and good reading habits are necessary for elderly people to prevent cognitive decline. In the present study, participants who engaged in a greater number of community activities and performed more housework were less likely to experience cognitive decline. Many studies have shown that social engagement may help decrease the risk of further cognitive decline [44], and a deterioration in the ability to perform housework is a potentially important indicator of an evolving cognitive impairment in some older people [46]. The social groups and family of older individuals should encourage and support their participation in as many social and family activities as possible. In our study, a higher monthly income was also a protective factor that prevented cognitive decline in elderly people. This result requires more in-depth investigations in future studies for confirmation.

Cognitive training may improve the cognitive function of patients with MCI and may decrease the rate at which MCI progresses to dementia [44, 47, 48] or AD [49]. More than 50.00% of people with MCI subsequently develop dementia [14]. Currently, cognitive training is frequently used in patients with MCI, and it generally includes physical activity training and mental training. This study is the first to investigate the efficacy of a cognitive training intervention in elderly individuals from three communities in Nanning, Guangxi, China. A substantial proportion of individuals with MCI revert to normal cognition in follow-up studies with no intervention measures [35]. In our study, significantly higher improvements to normal levels were observed after cognitive training in the intervention group (31.48%, 17/54) than in the control group (1.69%, 1/59); these changes were even present after three months of intervention (23.73%, 14/59 versus 1.59%, 1/63). Therefore, although a substantial proportion of individuals with MCI in the control group improved to normal levels, we still recommend cognitive training as the best and most effective method for improving the cognitive functions of patients with MCI.

According to some studies, cognitive training exerts beneficial effects on visuospatial/executive function, attention, language, delayed recall, and orientation in individuals with MCI [45, 50, 51]. In our study, cognitive function was improved in the intervention group after six months of cognitive training, but the control group had deteriorated after six months. The two-way repeated-measures ANOVA identified an effect of the interaction between the intervention time and cognitive training on the total MoCA score, but statistically significant effects of the intervention time (*p*=0.453) or grouping (*p*=0.050) alone on the total MoCA score were not observed. Perhaps we need to expand the sample size and intervention time to verify whether the intervention time or grouping affects cognitive function in future studies.

The present study has several limitations. First, because we used the MoCA-BJ to evaluate the function of elderly people, the participants were required to follow the investigators' commands. Elderly people who were illiterate or less educated might have experienced difficulty in comprehending the instructions and might not have followed the commands appropriately. These factors might have affected the MoCA scores. Second, as some participants were unable to insist on participating in the six-month cognitive training, an increase in the number of participants lost to follow-up was observed. This loss to follow-up might have influenced the accuracy of the analysis of the efficacy of cognitive training. A larger sample size might mitigate this problem. Finally, the study used the group training method for the intervention group, and all subjects were assembled in a room to deliver the training. Subjects with less education or other limitations might not have been comfortable in asking questions or clarifying any issues that they did not understand. The implementation of a smaller group based on the sociodemographic characteristics of the participants might have been a better approach. Future studies should focus on the limitations described above in the study planning process.

## 5. Conclusions

In conclusion, based on the findings of the current study, older age is associated with a cognitive decline. Factors that are more likely to protect against cognitive decline include a higher education level and monthly income, sufficient sleep time, regular physical exercise and reading habits, frequently engaging in community activities, and continuing to perform housework. Moreover, the cognitive training intervention is effective and may help decrease the deterioration of cognitive function in patients with MCI. The interaction between intervention time and cognitive training also significantly improves cognitive function.

## Figures and Tables

**Table 1:** MoCA scores recorded in the baseline survey (*N* = 2886).

Cognitive function	MoCA score (mean ± SD)
Total MoCA score	21.69 ± 4.64
Visuospatial/executive function	2.81 ± 1.34
Naming	2.57 ± 0.67
Attention	4.83 ± 1.33
Digit span (forward/backward)	1.79 ± 0.47
Vigilance (tapping)	0.58 ± 0.49
Serial 7 subtraction	2.45 ± 0.83
Language	1.64 ± 0.98
Sentence repetition	1.05 ± 0.75
Verbal fluency	0.59 ± 0.49
Abstraction	1.33 ± 0.76
Delayed recall	1.97 ± 1.47
Orientation	5.66 ± 0.82

**Table 2:** Distribution of MoCA scores according to demographic characteristics.

Characteristic	Sample (*N*)	MoCA score (Mean ± SD)	*t* test/one-way ANOVA		Spearman correlation analysis	
			*t*/*F* value	*p* value	Correlation coefficient (*r*)	*p* value
Total	2886	21.69 ± 4.64				
Gender			7.893	<0.001		
Male	1201	22.47 ± 4.16				
Female	1685	21.13 ± 4.89				
Age (years)			145.736	<0.001	−0.418	<0.001
60–64	284	24.02 ± 3.26				
65–69	1356	23.07 ± 3.71				
70–74	644	21.18 ± 4.07				
75–79	343	18.98 ± 4.99				
≧80	259	16.76 ± 5.68				
Education level (years of education)			265.461	<0.001	0.491	<0.001
Illiteracy (0 years)	306	15.13 ± 4.96				
Primary school (6 years)	934	20.72 ± 4.16				
Middle school (9 years)	836	22.83 ± 3.52				
High school (12 years)	508	23.99 ± 3.34				
College (≧13 years)	302	24.32 ± 2.95				
Monthly income ($)			86.302	<0.001	0.361	<0.001
0	399	18.36 ± 5.58				
1–148	351	19.61 ± 4.83				
149–297	596	21.48 ± 4.26				
298–446	904	22.74 ± 3.91				
447–595	364	23.63 ± 3.59				
≧596	272	23.63 ± 3.35				
Sleep time (hours/day)			29.271	<0.001	0.132	<0.001
<4	129	17.88 ± 5.34				
<6	1394	21.55 ± 4.66				
<8	1097	22.09 ± 4.36				
≧8	266	22.65 ± 4.41				
Exercise time (hours/week)			85.422	<0.001	0.275	<0.001
0	502	18.74 ± 5.33				
<1	1650	21.95 ± 4.40				
≧1	637	23.10 ± 3.74				
≧2	97	23.29 ± 3.41				
Reading (times/week)			114.360	<0.001	0.318	<0.001
0	1564	20.35 ± 4.89				
1-2	707	22.93 ± 3.70				
3–5	270	23.79 ± 3.77				
≥6	345	23.59 ± 3.81				
Community activities (times/week)			15.484	<0.001	0.129	<0.001
0	776	20.71 ± 5.24				
1–3	1249	21.71 ± 4.47				
4–6	627	22.60 ± 4.24				
7–9	198	22.43 ± 3.94				
≧10	36	22.31 ± 3.19				
Housework (times/week)			13.404	<0.001	0.082	<0.001
0	287	19.80 ± 5.45				
1-2	639	21.90 ± 4.24				
3–5	546	21.75 ± 4.24				
≧6	1414	21.96 ± 4.71				

**Table 3:** Basic information of the intervention and control groups.

Category	Sample (*N*)		Loss to follow-up (*N*)		Age (mean ± SD) (years)	
	Intervention group	Control group	Intervention group	Control group	Intervention group	Control group
Baseline						
Gender						
Male	27	35	NA	NA	67.70 ± 4.44	69.09 ± 4.27
Female	43	35	NA	NA	68.81 ± 4.04	68.29 ± 4.27
Total	70	70	NA	NA	68.39 ± 4.21	68.69 ± 4.26
Medium-term (follow-up at 3 months)						
Gender						
Male	22	32	5	3	67.82 ± 4.67	69.25 ± 4.41
Female	37	31	6	4	68.54 ± 4.23	68.19 ± 4.47
Total	59	63	11	7	68.27 ± 4.37	68.73 ± 4.43
Final-term (follow-up at 6 months)						
Gender						
Male	20	29	7	6	67.95 ± 4.55	69.21 ± 4.59
Female	34	30	9	5	68.41 ± 4.22	68.37 ± 4.44
Total	54	59	16	11	68.24 ± 4.31	68.78 ± 4.49

NA: not applicable.

**Table 4:** MoCA scores of the intervention and control groups.

Cognitive function	MoCA score (mean ± SD)		
	Baseline (before the intervention)	Medium-term (follow-up at 3 months)	Final-term (follow-up at 6 months)
Intervention group			
Total MoCA score	19.77 ± 2.24	20.64 ± 2.49	21.09 ± 2.20
Visuospatial/executive function	2.61 ± 1.13	2.71 ± 0.97	2.72 ± 0.81
Naming	2.26 ± 0.74	2.29 ± 0.70	2.33 ± 0.55
Attention	4.49 ± 1.11	4.78 ± 1.04	4.96 ± 0.82
Digit span (forward/backward)	1.73 ± 0.51	1.76 ± 0.43	1.70 ± 0.46
Vigilance (tapping)	0.39 ± 0.49	0.58 ± 0.50	0.70 ± 0.46
Serial 7 subtraction	2.37 ± 0.71	2.44 ± 0.73	2.56 ± 0.60
Language	1.50 ± 0.81	1.53 ± 0.75	1.56 ± 0.63
Sentence repetition	0.97 ± 0.66	0.73 ± 0.67	0.72 ± 0.60
Verbal fluency	0.53 ± 0.50	0.80 ± 0.41	0.83 ± 0.38
Abstraction	1.10 ± 0.73	1.14 ± 0.63	1.15 ± 0.60
Delayed recall	1.33 ± 1.06	1.64 ± 0.87	1.69 ± 0.70
Orientation	5.53 ± 0.76	5.64 ± 0.58	5.74 ± 0.44
Control group			
Total MoCA score	20.41 ± 2.10	19.40 ± 2.57	19.17 ± 2.57
Visuospatial/executive function	2.83 ± 0.87	2.68 ± 0.78	2.64 ± 0.74
Naming	2.19 ± 0.77	2.21 ± 0.68	2.22 ± 0.67
Attention	4.61 ± 0.92	4.49 ± 1.16	4.34 ± 1.14
Digit span (forward/backward)	1.83 ± 0.38	1.65 ± 0.48	1.59 ± 0.50
Vigilance (tapping)	0.37 ± 0.49	0.48 ± 0.50	0.42 ± 0.50
Serial 7 subtraction	2.41 ± 0.67	2.37 ± 0.75	2.32 ± 0.71
Language	1.59 ± 0.81	1.32 ± 0.78	1.22 ± 0.72
Sentence repetition	1.06 ± 0.68	0.68 ± 0.59	0.59 ± 0.59
Verbal fluency	0.53 ± 0.50	0.63 ± 0.49	0.63 ± 0.49
Abstraction	1.11 ± 0.83	0.95 ± 0.63	0.95 ± 0.63
Delayed recall	1.46 ± 0.93	1.33 ± 1.00	1.31 ± 0.81
Orientation	5.71 ± 0.64	5.51 ± 0.78	5.59 ± 0.62

**Table 5:** Results of the two-way repeated-measures ANOVA of the MoCA scores of the intervention and control groups after three and six months of cognitive training.

Cognitive function	Source	Degrees of freedom (df)	*F* value	*p* value	Partial eta-squared (*ηp*^2^)
Total MoCA score	Cognitive training	1.000	3.923	0.050	0.034
	Time	1.731	0.757	0.453	0.007
	Time *∗* cognitive training	1.731	46.459	<0.001	0.295
Visuospatial/executive function	Cognitive training	1.000	0.106	0.745	0.001
	Time	1.448	0.040	0.917	0.000
	Time *∗* cognitive training	1.448	1.680	0.196	0.015
Naming	Cognitive training	1.000	0.524	0.471	0.005
	Time	1.318	1.255	0.276	0.011
	Time *∗* cognitive training	1.318	0.192	0.730	0.002
Attention	Cognitive training	1.000	3.112	0.080	0.027
	Time	1.571	0.574	0.524	0.005
	Time *∗* cognitive training	1.571	7.995	0.001	0.067
Digit span (forward/backward)	Cognitive training	1.000	0.425	0.516	0.004
	Time	1.623	3.162	0.055	0.028
	Time *∗* cognitive training	1.623	3.070	0.059	0.027
Vigilance (tapping)	Cognitive training	1.000	3.459	0.066	0.030
	Time	1.512	8.019	0.001	0.067
	Time *∗* cognitive training	1.512	3.462	0.046	0.030
Serial 7 subtraction	Cognitive training	1.000	1.120	0.292	0.010
	Time	1.793	0.066	0.920	0.001
	Time *∗* cognitive training	1.793	3.061	0.055	0.027
Language	Cognitive training	1.000	1.407	0.238	0.013
	Time	1.499	2.105	0.138	0.019
	Time *∗* cognitive training	1.499	5.581	0.009	0.048
Sentence repetition	Cognitive training	1.000	0.079	0.779	0.001
	Time	1.546	20.832	<0.001	0.158
	Time *∗* cognitive training	1.546	1.942	0.157	0.017
Verbal fluency	Cognitive training	1.000	2.524	0.115	0.022
	Time	1.252	9.707	0.001	0.080
	Time *∗* cognitive training	1.252	3.531	0.053	0.031
Abstraction	Cognitive training	1.000	1.407	0.238	0.013
	Time	1.306	0.290	0.653	0.003
	Time *∗* cognitive training	1.306	2.422	0.113	0.021
Delayed recall	Cognitive training	1.000	2.342	0.129	0.021
	Time	1.637	1.022	0.349	0.009
	Time ∗ cognitive training	1.637	3.422	0.044	0.030
Orientation	Cognitive training	1.000	0.001	0.977	0.000
	Time	1.817	0.445	0.623	0.004
	Time *∗* cognitive training	1.817	4.633	0.013	0.040

**Table 6:** Comparison of the efficacy of the intervention after three and six months of cognitive training.

Category	The percentage of participants who improved to normal levels		The percentage of participants who progressed to dementia		The percentage of participants whose condition did not change		Chi-square (*χ*^2^) value	*p* value
	Normal (*N*)	Percentage (%)	Dementia (*N*)	Percentage (%)	MCI (*N*)	Percentage (%)		
Medium-term (follow-up at 3 months)								
Group							17.126	<0.001
Intervention (*N* = 59)	14	23.73	0	0.00	45	76.27		
Control (*N* = 63)	1	1.59	1	1.59	61	96.83		
Total (*N* = 122)	15	12.30	1	0.82	106	86.89		
Final-term (follow-up at 6 months)								
Group							24.711	<0.001
Intervention (*N* = 54)	17	31.48	0	0.00	37	68.52		
Control (*N* = 59)	1	1.69	3	5.08	55	93.22		
Total (*N* = 113)	18	15.93	3	2.65	92	81.42		

## Data Availability

The data used to support the findings of this study are available from the corresponding author upon request. The readers can contact Professor Li Yang via e-mail (yangli8290@hotmail.com) to obtain data.

## References

[1] Rao D., Luo X., Tang M. (2018). Prevalence of mild cognitive impairment and its subtypes in community-dwelling residents aged 65 years or older in Guangzhou, China. *Archives of Gerontology and Geriatrics*.

[2] Klimova B., Maresova P. (2017). Computer-based training programs for older people with mild cognitive impairment and/or dementia. *Frontiers in Human Neuroscience*.

[3] Matyas N., Auer S., Gisinger C. (2017). Continuing education for the prevention of mild cognitive impairment and Alzheimer’s-type dementia: a systematic review protocol. *Systematic Reviews*.

[4] Flak M. M., Hernes S. S., Skranes J., Løhaugen G. C. (2014). The memory aid study: protocol for a randomized controlled clinical trial evaluating the effect of computer-based working memory training in elderly patients with mild cognitive impairment (MCI). *Trials*.

[5] Hugo J., Ganguli M. (2014). Dementia and cognitive impairment. *Clinics in Geriatric Medicine*.

[6] Chang C.-F., Yang R.-J., Chang S.-F., Chou Y. H., Huang E.-W. (2017). The effects of quality of life and ability to perform activities of daily living on mild cognitive impairment in older people living in publicly managed congregate housing. *Journal of Nursing Research*.

[7] Knopman D. S., Petersen R. C. (2014). Mild cognitive impairment and mild dementia: a clinical perspective. *Mayo Clinic Proceedings*.

[8] Cecato J. F., Martinelli J. E., Izbicki R., Yassuda M. S., Aprahamian I. (2016). A subtest analysis of the Montreal cognitive assessment (MoCA): which subtests can best discriminate between healthy controls, mild cognitive impairment and Alzheimer’s disease?. *International Psychogeriatrics*.

[9] Alkhunizan M., Alkhenizan A., Basudan L. (2018). Prevalence of mild cognitive impairment and dementia in Saudi arabia: a community-based study. *Dementia and Geriatric Cognitive Disorders Extra*.

[10] Ma F., Wu T., Zhao J. (2016). Prevalence of mild cognitive impairment and its subtypes among Chinese older adults: role of vascular risk factors. *Dementia and Geriatric Cognitive Disorders*.

[11] Ding D., Zhao Q., Guo Q. (2015). Prevalence of mild cognitive impairment in an urban community in China: a cross-sectional analysis of the Shanghai Aging Study. *Alzheimer’s & Dementia*.

[12] Mirza S. S., Ikram M. A., Bos D., Mihaescu R., Hofman A., Tiemeier H. (2017). Mild cognitive impairment and risk of depression and anxiety: a population-based study. *Alzheimer’s & Dementia*.

[13] Bae J. B., Kim Y. J., Han J. W. (2015). Incidence of and risk factors for Alzheimer’s disease and mild cognitive impairment in Korean elderly. *Dementia and Geriatric Cognitive Disorders*.

[14] Behrman S., Valkanova V., Allan C. L. (2017). Diagnosing and managing mild cognitive impairment. *Practitioner*.

[15] Lebedeva E., Gallant S., Tsai C. E., Koski L. (2015). Improving the measurement of cognitive ability in geriatric patients. *Dementia and Geriatric Cognitive Disorders*.

[16] Coyle H., Traynor V., Solowij N. (2015). Computerized and virtual reality cognitive training for individuals at high risk of cognitive decline: systematic review of the literature. *The American Journal of Geriatric Psychiatry*.

[17] Hill N. T. M., Mowszowski L., Naismith S. L., Chadwick V. L., Valenzuela M., Lampit A. (2017). Computerized cognitive training in older adults with mild cognitive impairment or dementia: a systematic review and meta-analysis. *American Journal of Psychiatry*.

[18] Reijnders J., van Heugten C., van Boxtel M. (2013). Cognitive interventions in healthy older adults and people with mild cognitive impairment: a systematic review. *Ageing Research Reviews*.

[19] Simon S. S., Yokomizo J. E., Bottino C. M. C. (2012). Cognitive intervention in amnestic Mild Cognitive Impairment: a systematic review. *Neuroscience & Biobehavioral Reviews*.

[20] Belleville S. (2008). Cognitive training for persons with mild cognitive impairment. *International Psychogeriatrics*.

[21] Gates N. J., Sachdev P. S., Fiatarone Singh M. A., Valenzuela M. (2011). Cognitive and memory training in adults at risk of dementia: a systematic review. *BMC Geriatrics*.

[22] Kurz A. F., Leucht S., Lautenschlager N. T. (2011). The clinical significance of cognition-focused interventions for cognitively impaired older adults: a systematic review of randomized controlled trials. *International Psychogeriatrics*.

[23] Huckans M., Hutson L., Twamley E., Jak A., Kaye J., Storzbach D. (2013). Efficacy of cognitive rehabilitation therapies for mild cognitive impairment (MCI) in older adults: working toward a theoretical model and evidence-based interventions. *Neuropsychology Review*.

[24] Vaccaro M. G., Izzo G., Ilacqua A. (2019). Characterization of the effects of a six-month dancing as approach for successful aging. *International Journal of Endocrinology*.

[25] Yu J., Li J., Huang X. (2012). The Beijing version of the Montreal Cognitive Assessment as a brief screening tool for mild cognitive impairment: a community-based study. *BMC Psychiatry*.

[26] Kaya Y., Aki O. E., Can U. A., Derle E., Kibaroğlu S., Barak A. (2014). Validation of montreal cognitive assessment and discriminant power of montreal cognitive assessment subtests in patients with mild cognitive impairment and alzheimer dementia in Turkish population. *Journal of Geriatric Psychiatry and Neurology*.

[27] Guaita A., Vaccaro R., Davin A. (2015). Influence of socio-demographic features and apolipoprotein E epsilon 4 expression on the prevalence of dementia and cognitive impairment in a population of 70–74-year olds: the InveCe.Ab study. *Archives of Gerontology and Geriatrics*.

[28] Anderson-Hanley C., Stark J., Wall K. M. (2018). The interactive physical and cognitive exercise system (iPACES™;): effects of a 3-month in-home pilot clinical trial for mild cognitive impairment and caregivers. *Clinical Interventions in Aging*.

[29] Petersen R. C., Smith G. E., Ivnik R. J. (1995). Apolipoprotein E status as a predictor of the development of Alzheimer’s disease in memory-impaired individuals. *JAMA: The Journal of the American Medical Association*.

[30] Clarnette R., O’Caoimh R., Antony D. N., Svendrovski A., Molloy D. W. (2017). Comparison of the quick mild cognitive impairment (qmci) screen to the montreal cognitive assessment (MoCA) in an Australian geriatrics clinic. *International Journal of Geriatric Psychiatry*.

[31] Bosco A., Spano G., Caffò A. O. (2017). Italians do it worse. Montreal Cognitive Assessment (MoCA) optimal cut-off scores for people with probable Alzheimer’s disease and with probable cognitive impairment. *Aging Clinical and Experimental Research*.

[32] Lu J., Li D., Li F. (2011). Montreal cognitive assessment in detecting cognitive impairment in Chinese elderly individuals: a population-based study. *Journal of Geriatric Psychiatry and Neurology*.

[33] Hu J., Xiao-min W., Kai-yong H. (2019). Study on prevalence of and influencing factors of mild cognitive impairment among elderly people in communities of Nanning. *Chinese Journal of Disease Control & Prevention*.

[34] Soleimani R., Shokrgozar S., Fallahi M. (2018). An investigation into the prevalence of cognitive impairment and the performance of older adults in Guilan province. *Journal of Medicine and Life*.

[35] Gao S., Unverzagt F. W., Hall K. S. (2014). Mild cognitive impairment, incidence, progression, and reversion: findings from a community-based cohort of elderly African Americans. *The American Journal of Geriatric Psychiatry*.

[36] Alhurani R. E., Vassilaki M., Aakre J. A. (2016). Decline in weight and incident mild cognitive impairment. *JAMA Neurology*.

[37] Buscemi S., Di Pasquale V., Buscemi C., Piccoli T., Giordano C. (2017). Factors associated with mild cognitive impairment in a population-based cohort. *European Journal of Internal Medicine*.

[38] Ozer S., Young J., Champ C., Burke M. (2016). A systematic review of the diagnostic test accuracy of brief cognitive tests to detect amnestic mild cognitive impairment. *International Journal of Geriatric Psychiatry*.

[39] Kandiah N., Zhang A., Cenina A. R., Au W. L., Nadkarni N., Tan L. C. (2014). Montreal Cognitive Assessment for the screening and prediction of cognitive decline in early Parkinson’s disease. *Parkinsonism & Related Disorders*.

[40] Oren N., Yogev-Seligmann G., Ash E. (2014). The Montreal Cognitive Assessment in cognitively-intact elderly: a case for age-adjusted cutoffs. *Journal of Alzheimer’s Disease*.

[41] Chu L.-W., Ng K. H., Law A. C., Lee A. M., Kwan F. (2015). Validity of the Cantonese Chinese montreal cognitive assessment in southern Chinese. *Geriatrics & Gerontology International*.

[42] Tumas V., Borges V., Ballalai-Ferraz H. (2016). Some aspects of the validity of the Montreal Cognitive Assessment (MoCA) for evaluating cognitive impairment in Brazilian patients with Parkinson’s disease. *Dementia & Neuropsychologia*.

[43] da Silva R. A. P. C. (2015). Sleep disturbances and mild cognitive impairment: a review. *Sleep Science*.

[44] Langa K. M., Levine D. A. (2014). The diagnosis and management of mild cognitive impairment. *JAMA*.

[45] Suzuki H., Kuraoka M., Yasunaga M. (2014). Cognitive intervention through a training program for picture book reading in community-dwelling older adults: a randomized controlled trial. *BMC Geriatrics*.

[46] Jiang C., Xu Y. (2014). The association between mild cognitive impairment and doing housework. *Aging & Mental Health*.

[47] Okamura H., Otani M., Shimoyama N., Fujii T. (2018). Combined exercise and cognitive training system for dementia patients: a randomized controlled trial. *Dementia and Geriatric Cognitive Disorders*.

[48] Petersen R. C., Lopez O., Armstrong M. J. (2018). Practice guideline update summary: mild cognitive impairment. *Neurology*.

[49] Kirova A. M., Bays R. B., Lagalwar S. (2015). Working memory and executive function decline across normal aging, mild cognitive impairment, and Alzheimer’s disease. *BioMed Research International*.

[50] Liu X. Y., Li L., Xiao J. Q. (2016). Cognitive training in older adults with mild cognitive impairment. *Biomedical and Environmental Sciences*.

[51] Young D. K.-w., Ng P. Y.-n., Kwok T., Cheng D. (2017). The effects of holistic health group interventions on improving the cognitive ability of persons with mild cognitive impairment: a randomized controlled trial. *Clinical Interventions in Aging*.

